# *Entamoeba histolytica*-Induced Mucin Exocytosis Is Mediated by VAMP8 and Is Critical in Mucosal Innate Host Defense

**DOI:** 10.1128/mBio.01323-17

**Published:** 2017-10-03

**Authors:** Steve Cornick, France Moreau, Herbert Y. Gaisano, Kris Chadee

**Affiliations:** a Department of Microbiology, Immunology and Infectious Diseases, Snyder Institute for Chronic Diseases, University of Calgary, Calgary, Alberta, Canada; b Departments of Medicine and Physiology, University of Toronto, Toronto, Ontario, Canada; Sequella, Inc.

**Keywords:** *Entamoeba histolytica*, exocytosis, goblet cells, gut inflammation, innate immunity, mucin, pathogenesis

## Abstract

Intestinal mucus secretion is critical in maintaining mucosal host defense against a myriad of pathogens by preventing direct association with the epithelium. *Entamoeba histolytica* specifically binds colonic MUC2 mucin and also induces potent hypersecretion from goblet cells; however, characterization of the nature of the mechanisms controlling mucus release remains elusive. In this report, we identify vesicle SNARE vesicle-associated membrane protein 8 (VAMP8) present on mucin granules as orchestrating regulated exocytosis in human goblet cells in response to the presence of *E. histolytica*. VAMP8 was specifically activated during *E. histolytica* infection, and ablation of VAMP8 led to impaired mucin secretion. As a consequence, loss of VAMP8 increased *E. histolytica* adherence to epithelial cells associated with enhanced cell death through apoptosis characterized by caspase 3 and 9 cleavages and DNA fragmentation. With the mucosal barrier compromised in *Vamp8*^−/−^ animals, *E. histolytica* induced an aggressive proinflammatory response with elevated levels of interleukin-1 alpha (IL-1α), IL-1β, and tumor necrosis factor alpha (TNF-α) secretion. This report is the first to characterize regulated mucin exocytosis in intestinal goblet cells in response to a pathogen and the downstream consequences of improper mucin secretion in mucosal barrier defense.

## IMPORTANCE

The intestinal tract is exposed to countless substances and pathogens, and yet homeostasis is maintained, in part by the mucus layer that houses the microbiota and spatially separates potential threats from the underlying single layer of epithelium. Despite the critical role of mucus in innate host defense, characterization of the mechanisms by which mucus is secreted from specialized goblet cells in the gut remains elusive. Here, we describe the machinery that regulates mucus secretion as well as the consequence during infection with the colonic pathogen *Entamoeba histolytica*. Abolishment of the key machinery protein VAMP8 abrogated mucus release in cultured human colonic goblet cells and during *E. histolytica* infection in *Vamp8*^−/−^ mice, which showed enhanced amoeba contact and killing of epithelial cells, triggering a potent proinflammatory response. This report highlights the importance of the VAMP8 secretory machinery in facilitating mucus release from intestinal goblet cells and the dire consequences that occur during disease pathogenesis if these pathways are not functional.

## INTRODUCTION

The gastrointestinal tract faces countless luminal threats, including those presented by noxious substances, commensal bacteria, and/or pathogens. The first line of innate host defense is the intestinal mucus layer that spatially separates these threats from the single layer of epithelial cells tasked with nutrient absorption (1). In the colon, the mucus layer is composed of an inner firmly adherent layer that is impenetrable to microbes and a looser outer layer that is heavily colonized with indigenous bacteria (2). Owing to its composition, the mucus layer acts a decoy to sustain biofilm growth of commensal bacteria due to the primary component (MUC2 mucin) being extensively glycosylated (3). MUC2 is a high-molecular-weight polymeric protein with extensive O-linked galactose, N-acetylgalactosamine, and N-acetylglucosamine components with terminal fucose and sialic acid glycans (4, 5). In addition to microbes binding to the mucus layer via lectin interactions, it also serves as a food source and is readily degraded (6). Replenishment and maintenance of the mucus layer are facilitated by the goblet cells that produce and store MUC2 within secretory granules. Mucus is secreted constitutively to maintain epithelial barrier function and is inducible when goblet cells sense a threat such as that presented by an invading pathogen (7, 8). Loss of the protective roles of mucin, such as in *Muc2*^−/−^ mice, leads to epithelial distress, tight junction permeability, and a proinflammatory response elicited by the host immune system as a secondary defense mechanism for protection from luminal threats (9, 10). Given the immense importance of the mucus layer in maintaining homeostasis, little is known about the mechanism of mucin release from intestinal goblet cells. Historically, numerous studies have alluded to exocytosis as a likely candidate for mucin secretion (11, 12).

Classical exocytosis involves coordination of soluble NSF (N-ethylmaleimide-sensitive factor) attachment protein receptors, SNAREs, to carry out vesicle-membrane fusion events. R-SNAREs, predominantly vesicle-associated membrane proteins (VAMP) present on vesicles, bind to Q_abc_-SNARE complexes present on the plasma membrane following a stimulatory signal, allowing exocytosis of vesicle content (13). These Q_abc_ complexes are typically composed of the synaptosome-associated proteins (SNAP) and syntaxins whereby many SNARE chaperones mediate this core complex formation (14). Previously work on induced mucin exocytosis demonstrated a high dependency on kinases, specifically, protein kinase C (PKC), in facilitating mucin release in response to the mucosal pathogen *Entamoeba histolytica* (15). This protozoan parasite is responsible for amoebiasis and significant mortality in developing countries. *E. histolytica* has a unique interaction with mucin, allowing adhesion, degradation, and secretion of mucin. First, *E. histolytica* specifically binds to glycans in MUC2 through a 170-kDa adherence Gal/GalNAc lectin (6). Second, *E. histolytica* possesses specific cysteine proteases (CP5) that target MUC2 for degradation, allowing penetration of the mucus layer (16). This virulence factor is also critical in inducing mucus hypersecretion and depletion by binding to host integrins and inducing a signaling cascade involving focal adhesion kinase (FAK)/phosphatidylinositol 3-kinase (PI3K)/PKCδ (15). Breakdown of these protective mechanisms is fundamental in *E. histolytica* pathogenesis and leads to direct cytolysis of epithelial cells. Amoebic-induced cell death may occur either through trogocytosis, in which small bits of the plasma membrane of the host cell are ingested, or through the activity of the amoebapore. With the epithelial barrier compromised, intestinal macrophages then mount a robust proinflammatory response through activation of the NLRP3 inflammasome, leading to interleukin-1 alpha (IL-1α) and IL-1β release (17). The mechanisms governing mucin exocytosis in goblet cells remain to be elucidated despite its being a critical component in the innate host defense against pathogens. Here, we describe for the first time how mucin secretion for intestinal goblet cells follows classical exocytosis and interrogate the R-SNARE VAMP8 as the critical vesicle SNARE in facilitating its release in response to a pathogen. Loss of VAMP8 led to impaired mucin secretion, culminating in increased adherence of *E. histolytica* to epithelial cells. This ultimately led to apoptosis of host cells and a subsequent proinflammatory response, exacerbating *E. histolytica* pathogenesis.

## RESULTS

### VAMP8 facilitates mucin exocytosis and is specifically activated by *E. histolytica*.

**FIG 1:**
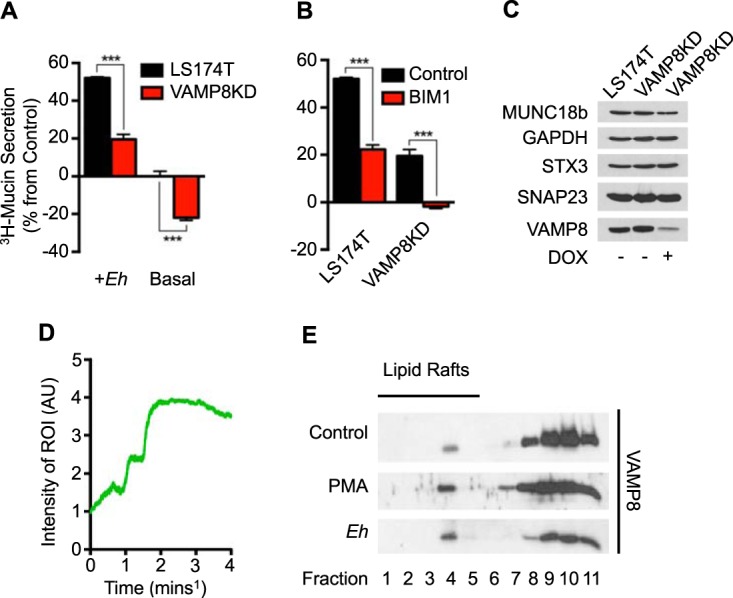
VAMP8 facilitates mucin exocytosis and is specifically activated by *E. histolytica in vitro*. (A) shRNA lentivirus-inducible LS174T cells were placed in contact with *E. histolytica* (*Eh*) for 2 h and [^3^H]glucosamine-labeled mucin secretion quantified. VAMP8KD cells were compared to uninduced cells, where less mucin secretion was seen in VAMP8KD cells. (B) PKC inhibition by the broad PKC inhibitor bisindolylmaleimide I (BIM1) was hindered in both uninduced and VAMP8KD LS174T cells. (C) shRNA KD of VAMP8 was confirmed by Western blotting, where LS174T and uninduced VAMP8 cells displayed normal VAMP8 expression, in contrast to doxycycline (DOX; 2 µg/ml)-induced VAMP8KD cells, which had >60% less VAMP8. The expression of other SNAREs was not affected by VAMP8KD. GAPDH, glyceraldehyde-3-phosphate dehydrogenase. (D) VAMP8 exocytosis was monitored by live-cell imaging using pHluorin, where the area colocalizing to MUC2 showed a time-dependent increase in GFP signal following infection with *E. histolytica*. Representative tracing from one ROI is displayed. AU, arbitrary units. (E) Indicative of classical exocytosis, VAMP8 translocated to lipid raft domains within the plasma membrane similarly to the mucin secretagogue PMA following infection with *E. histolytica*. ***, *P* < 0.001.

To understand the mechanism of robust mucin release from intestinal goblet cells following *E. histolytica* contact, we first utilized LS174T cells, which take on a goblet cell phenotype (18). We have previously shown that, following contact with *E. histolytica*, there is a signaling cascade that culminates in MARCKS phosphorylation (15). We observed the vesicle SNARE VAMP8 on the activated phospho-MARCKS-positive mucin granules, suggesting that VAMP8 participates in mucin exocytosis. To confirm this, VAMP8 was silenced in LS174T cells by the use of a lentivirus-inducible small hairpin RNA (shRNA). Upon doxycycline treatment, VAMP8-knockdown (KD) cells expressed TurboRFP, whereupon these cells were routinely subjected to fluorescence-activated cell sorter (FACS) analysis to obtain a highly silenced phenotype. In all experiments, uninduced LS174T cells that harbored the lentivirus construct were used and no knockdown of VAMP8 was observed. To elucidate a role for VAMP8 in facilitating mucin release, we metabolically labeled the glycans in mucin with [^3^H]glucosamine to monitor high-molecular-weight-mucin secretion by scintillation counting of the [^3^H]mucin. In accordance with our previous studies (19), *E. histolytica* induced robust secretion of mucin from controls at 2 h; however, VAMP8KD cells were significantly inhibited (Fig. 1A) with respect to their ability to release mucin. Additionally, basal mucin secretion in the absence of *E. histolytica* was also inhibited in VAMP8KD cells, suggesting that VAMP8 is critical in both agonist-induced and constitutive mucin secretion (Fig. 1A). Interestingly, the magnitudes of mucin secretion induced in response to *E. histolytica* compared to the basal level were analogous in LS174T and VAMP8KD cells, despite the damping of the absolute quantity of mucin released. This was likely due to incomplete knockdown of VAMP8 in LS174T cells, whereby constant magnitude changes indicated a lack of any compensatory mucin secretion mechanisms. To quantify signaling pathway specificity, the broad-scale PKC inhibitor bisindolylmaleimide I (BIM1) was used. BIM1 hindered mucin secretion in control cells by more than 50% and completely abrogated mucin secretion in VAMP8KD cells (Fig. 1B). At that dose and time point of *E. histolytica* infection, we routinely assay cell death to ensure that mucin release is truly the result of exocytosis and not of the release of cytoplasmic contents following cell death. By targeting two components of the mucin exocytosis machinery, VAMP8 and PKC, the secretion induced by *E. histolytica* could be completely blocked. The other SNARE proteins that we suspected of participating in exocytosis, specifically, SNAP23, syntaxin 3, and Munc18b, were not affected by VAMP8KD (Fig. 1C); however, aberrant expression of VAMP2 was often seen in VAMP8KD cells.

To assess VAMP8 activation, we used a reporter construct (pHluorin-VAMP8) that contains a pH-sensitive green fluorescent protein (GFP) within the cytoplasmic compartment of the mucin granule (20). Previous studies have shown that the tight packaging of mucin within the mucin granule is highly pH dependent and that low (~5.4) pH is critical in compacting mucin (21). When a mucin granule undergoes exocytosis, the luminal pH (~7.4) allows the unfolding of mucin and allows pHluorin to increase fluorescence. Using live-cell imaging of pHluorin-VAMP8 and MUC2CK-mRuby2 transfected cells, we observed colocalization of VAMP8 with MUC2^+^ granules within the cytoplasm in basal cells (see Movie S1 in the supplemental material) (22). Basally, these granules localize to areas in close proximity to the plasma membrane; however, exocytosis events are rare, as evidenced from a lack of VAMP-pHluorin fluorescence despite the occurrence of vesicle docking. However, following stimulation with *E. histolytica*, VAMP8 polarized to a central region within the cytoplasm surrounding a large MUC2^+^ organizing center (Movie S2). There was a 4-fold increase in VAMP8 fluorescence following treatment with *E. histolytica* at up to 40 min postinfection, and VAMP8 was observed in contact in the plasma membrane (Fig. 1D). Interestingly, the compartment containing mucin granules increased in size drastically following stimulation with *E. histolytica*, suggesting compound mucin exocytosis. This activation state of VAMP8 was prolonged; VAMP8-pHluorin reached peak fluorescence at 20 min following *E. histolytica* contact and continued facilitating compound exocytosis maximally up to 40 min. Although we observed a reduction in MUC2 fluorescence within the VAMP8 granule compartment, a result highly suggestive of MUC2 content emptying, we cannot ascertain whether or not this was due to photobleaching. To confirm activation of VAMP8 following *E. histolytica* treatment, we isolated lipid raft domains from control, phorbol myristate acetate (PMA)-, and *E. histolytica*-stimulated LS174T cells. Previous studies have shown that upon exocytosis, various SNARE components are localized to lipid raft domains (23). Given that VAMP8 does not normally associate with the plasma membrane basally due to its cytoplasmic localization, VAMP8 present in lipid raft domains served as a readout for participation in exocytosis. Indeed, control cells had very little VAMP8 present within lipid raft domains (fractions 4 to 5); however, upon stimulation with either the mucin secretagogue PMA or *E. histolytica*, VAMP8 polarized to lipid raft domains (Fig. 1E).

10.1128/mBio.01323-17.1 MOVIE S1  LS174T cells were transiently transfected with VAMP-pHluorin GFP (green) and MUC2CK-mRuby2 (red), and the plasma membrane was labeled with CellMask Far red (Blue). Untreated LS174T cells did not localize VAMP8 to an organizing center and instead colocalized VAMP8 to MUC2 granules. Download MOVIE S1, MOV file, 0.4 MB. Copyright © 2017 Cornick et al. 2017 Cornick et al. This content is distributed under the terms of the Creative Commons Attribution 4.0 International license.

10.1128/mBio.01323-17.2 MOVIE S2  LS174T cells were transiently transfected with VAMP-pHluorin GFP (green) and MUC2CK-mRuby2 (red), and the plasma membrane was labeled with CellMask Far red (Blue) and infected with CellTracker Blue *E. histolytica* (magenta) and imaged for up to 40 min. Stimulated LS174T polarized VAMP8 was localized to a mucin-organizing center following *E. histolytica* contact, and VAMP8 fluorescence was drastically increased. Download MOVIE S2, MOV file, 1.4 MB. Copyright © 2017 Cornick et al. 2017 Cornick et al. This content is distributed under the terms of the Creative Commons Attribution 4.0 International license.

**FIG 2:**
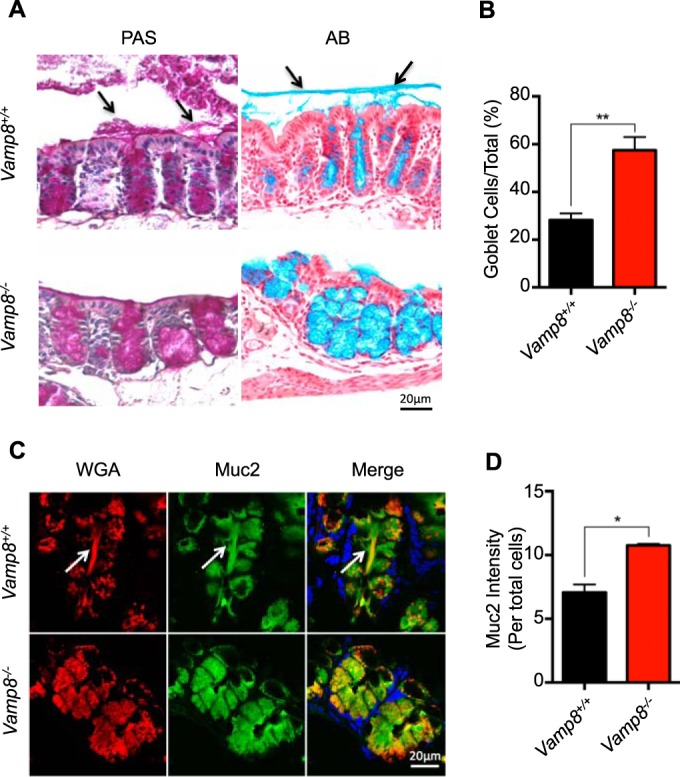
Vamp8 facilitates mucin exocytosis by *E. histolytica in vivo*. (A) In a colonic loop model of infection with *E. histolytica*, *Vamp8*^−/−^ animals lacked periodic acid Schiff (PAS)-positive and alcian blue (AB)-positive mucus secretion within the lumen. (B) Quantification of Muc2^+^ goblet cells following infection with *E. histolytica* in colonic loops was performed by analyzing at least 10 histological sections using high-power microscopy (40× objective) where *Vamp8*^−/−^ mice showed significantly more goblet cells than *Vamp*^*+/+*^ littermates. (C) Unlike *Vamp*^*+/+*^ colonic loops infected with *E. histolytica*, *Vamp8*^−/−^ colonic loops lacked WGA and Muc2^+^ mucin streaming out of colonic crypts (arrows) and displayed increased levels of mucin retained within the epithelium. (D) Quantification of Muc2^+^ cells following infection with *E. histolytica* in colonic loops was performed by analyzing confocal images. *Vamp8*^−/−^ mice displayed more Muc2^+^ mucin retained within the goblet cells indicative of improper goblet cell exocytosis and increased goblet cell numbers compared to *Vamp*^*+/+*^ littermates. **, *P* < 0.01; *, *P* < 0.05.

To validate these finding *in vivo*, we performed colonic loop infections with *E. histolytica* in *Vamp8*^−/−^ and *Vamp*^*+/+*^ littermate controls (24). Following 3 h of infection, tissues were fixed in Carnoy's fixative to preserve the mucus layer. In *Vamp*^*+/+*^ littermates, periodic acid Schiff-positive (PAS^+^) and alcian blue-positive (AB^+^) mucus secretion was observed in the surface epithelium and in the lumen of *E. histolytica*-infected loops (black arrows), whereas mucus secretion was absent in *Vamp8*^−/−^ littermates (Fig. 2A) and mucin granules were entirely retained in the goblet cells. Notably, *Vamp8*^−/−^ animals displayed aberrant granule morphology characterized by the presence of swollen goblet cells and increased numbers of goblet cells (Fig. 2B). In contrast to the *Vamp8*^*+/+*^ loops, where wheat germ agglutinin (WGA) and Muc2^+^ mucin were observed streaming out of colonic crypts (arrows), *Vamp8*^−/−^ animals lacked this secretory event (Fig. 2C). In particular, mucin was retained entirely within the swollen goblet cells in *Vamp*^−/−^ animals (Fig. 2D). These results demonstrate that VAMP8 is critical in facilitating mucus secretion and is specifically activated upon interaction with *E. histolytica in vitro*.

### Lack of VAMP8 and mucin exocytosis increases *E. histolytica* contact with goblet cells.

**FIG 3:**
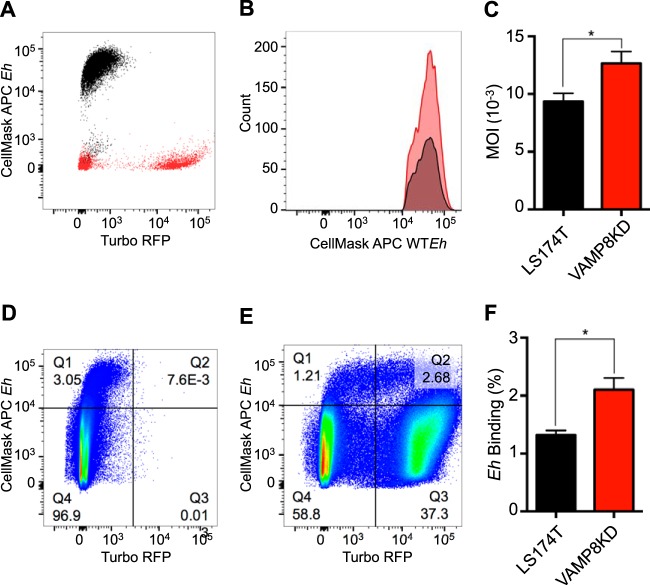
VAMP8KD increases *E. histolytica* contact with goblet cells due to aberrant mucin exocytosis. (A) LS174T cells were labeled with CFSE and infected with APC-Cellmask-labeled *E. histolytica* for 30 min, washed with PBS to eliminate nonadherent *E. histolytica*, dissociated using trypsin, and analyzed by flow cytometry. VAMP8KD cells expressed levels of TurboRFP that were directly proportional to the degree of gene silencing, where 40% of cells were knocked down. APC-*E. histolytica* scattered very differently, allowing quantification of *E. histolytica* with LS174T cells. (B) Uninduced and VAMP8KD cells were infected with *E. histolytica*, and the total amount of *E. histolytica* was analyzed by the use of APC-CellMask. (C) The multiplicity of infection (MOI) was higher in VAMP8KD cells as quantified by analysis of the ratio of the total amount of *E. histolytica* remaining after washing to the total amount of LS174T cells. (D and E) Uninduced LS174T cells (D) had less *E. histolytica* direct binding than VAMP8KD cells (E). Additionally, Q2 had more *E. histolytica* bound in TurboRFP^+^ VAMP8KD cells than in uninduced cells in the same well (Q1). (F) *E. histolytica* direct binding to TurboRFP^+^ VAMP8KD cells was also higher than in uninduced cells. *, *P* < 0.05.

Mucus secretion serves as the first line of innate defense during *E. histolytica* infection, and lack of the predominant vesicle SNARE VAMP8 abrogates this protective effect. Critical to *E. histolytica* pathogenesis, epithelial invasion results from *E. histolytica* cytopathic effects when direct contact occurs (25). We developed a flow cytometry protocol to assess *E. histolytica* binding on LS174T monolayers following 30 min of infection. To distinguish *E. histolytica* cells from epithelial cells, *E. histolytica* was labeled with allophycocyanin (APC)-CellMask and epithelial cells were labeled with carboxyfluorescein succinimidyl ester (CFSE). In addition to the results seen with dye-based methods, *E. histolytica* also scattered in a manner distinct from that seen with LS174T cells (Fig. 3A). VAMP8KD cells express TurboRFP, allowing monitoring of knockdown. Following 30 min of infection, nonadherent *E. histolytica* was washed with phosphate-buffered saline (PBS) and the remaining cells dissociated and were analyzed by flow cytometry. VAMP8KD cells had increased levels of adherent *E. histolytica* present compared to uninduced cells (Fig. 3B). The total amount of *E. histolytica* normalized to the total number of cells was quantified as the multiplicity of infection (MOI), and VAMP8KD cells were found to have more adherent *E. histolytica* cells (Fig. 3C). Uninduced cells that retain VAMP8 expression have a functional exocytosis machinery allowing mucin to act as a decoy for *E. histolytica* binding and thus led to less epithelial contact (Fig. 3D). In contrast, VAMP8KD TurboRFP^+^ cells showed a 2-fold increase in the level of *E. histolytica* cells that directly bound (quadrant 2 [Q2]), and the lack of mucin secretion also affected bystander cells that were not knocked down, with an increase in the total amount of *E. histolytica* (Fig. 3E). Furthermore, the amount of *E. histolytica* directly bound to VAMP8KD cells was higher than that seen with uninduced cells (Fig. 3F). Here, proper mucin secretion mediated by VAMP8 had consequences for protection against *E. histolytica*, allowing increased contact of *E. histolytica* with epithelial cells.

### Increased association of *E. histolytica* in cells lacking VAMP8 leads to cytopathic effects and apoptosis.

**FIG 4:**
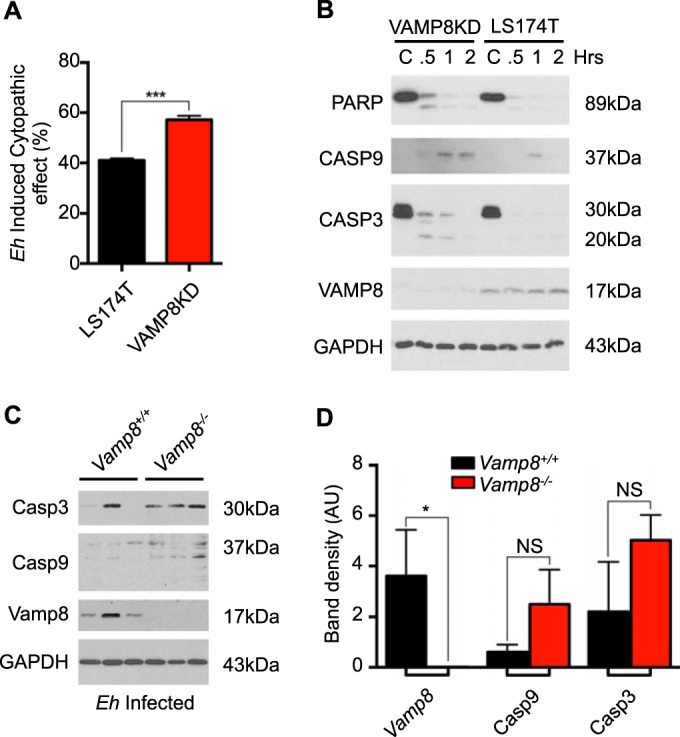
Lack of VAMP8 allows *E. histolytica* cytopathic effect and induced apoptosis. (A) LS174T cells were infected with *E. histolytica*, and the cytopathic effect was assessed by methylene blue staining after 4 h. VAMP8KD cells showed increased cytopathic killing of the monolayer compared to uninduced cells. (B) VAMP8 and classical apoptosis markers were assessed by Western blotting at various time points of infection with *E. histolytica*. VAMP8 expression was absent in induced cells; however, levels of cleaved caspase 3 (Casp3), caspase 9 (Casp9), and PARP were increased in a time-dependent manner with no spontaneous apoptosis detected in uninfected cells. C, control. (C) There were increased levels of cleaved caspase 3 and 9 in *E. histolytica*-infected *Vamp8*^−/−^ colonic loops, whereas cleavage was absent in *Vamp8*^*+/+*^ colonic loops. (D) Densitometry was performed to assess increased apoptosis in *Vamp8*^−/−^ loops. ***, *P* < 0.001; *, *P* < 0.05; NS, not significant.

**FIG 5:**
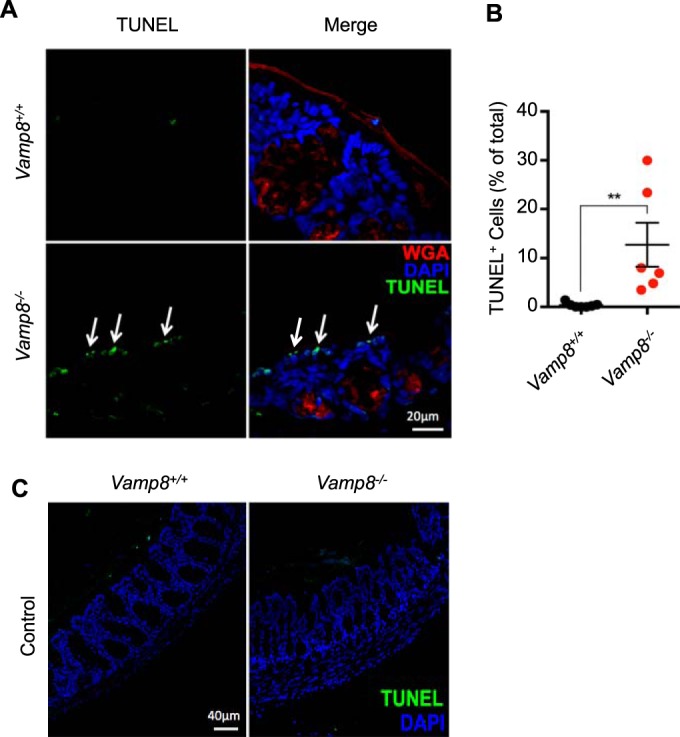
*Vamp8*^−/−^ leads to increased apoptosis *in vivo*. (A) *E. histolytica*-infected colonic loops were analyzed by TUNEL assay for DNA fragmentation, a hallmark of apoptosis, At 3 h postinfection, *Vamp8*^−/−^ mice showed increased TUNEL staining and apoptosis at the apical surface of the epithelium (arrows) compared to *Vamp*^*+/+*^ littermates. (B) Quantification of TUNEL^+^ cells within the epithelium was done, and the results were normalized to the total number of DAPI^+^ cells. *Vamp8*^−/−^ mice showed significantly greater numbers of TUNEL^+^ cells following infection with *E. histolytica*. (C) *Vamp8*^−/−^ did not show spontaneous basal apoptosis. Uninfected control tissues were stained for TUNEL analysis, and no differences between WT and *Vamp8*^−/−^ mice in the levels of TUNEL^+^ cells were observed. **, *P* < 0.01.

A prerequisite for *E. histolytica* pathogenesis is direct binding resulting in *E. histolytica* killing target cells and overcoming epithelial barrier function. In the absence of VAMP8 and subsequent mucin exocytosis, *E. histolytica* binds to epithelial cells and induces monolayer destruction (Fig. 4A). To emphasize this event, we used a MOI that was 5 times higher than that used in the secretion experiments and extended the infection period to 4 h to assess monolayer destruction. Apoptosis was assessed by Western blotting using the classical markers cleaved caspase 3 and 9 and poly (ADP-ribose) polymerase (PARP), where VAMP8KD cells showed increased cleavage that occurred in a time-dependent fashion compared to uninduced cells *in vitro* (Fig. 4B). These findings held true *in vivo* in the colonic loop model, where *Vamp8*^−/−^ animals showed increased caspase 3 and 9 cleavage at 3 h postinfection (Fig. 4C). Apoptosis in *Vamp8*^−/−^ animals was quantified by densitometry, where there was an increase in both caspase 3 and caspase 9 levels with no Vamp8 expression (Fig. 4D). To further confirm apoptosis *in vivo*, we performed a terminal deoxynucleotidyltransferase-mediated dUTP-biotin nick end labelling (TUNEL) assay to detect DNA fragmentation following infection with *E. histolytica*. *Vamp8*^−/−^ animals displayed TUNEL^+^ apoptotic cells at the apical surface of the epithelium following infection with *E. histolytica* (white arrows); however, *Vamp8*^*+/+*^-infected littermates did not (Fig. 5A). We routinely observed numerous TUNEL^+^ cells within the lumen of both *Vamp8*^*+/+*^ and *Vamp8*^−/−^ animals as a confirmation that the staining was specific. Abundance of TUNEL^+^ apoptotic cells was assessed on numerous confocal images and quantified, with the results showing that *Vamp8*^−/−^ animals had increased apoptosis compared to *Vamp8*^*+/+*^ littermates (Fig. 5B). This phenomenon was specific during acute infection, as *Vamp8*^*+/+*^ and *Vamp8*^−/−^ control tissues did not show any differences in TUNEL^+^ cells (Fig. 5C). These results demonstrate that VAMP8 mediates protection of the epithelium through facilitation of mucin secretion and that a lack of this protective effect leads to increased levels of *E. histolytica* cytopathic effects.

### Proinflammatory responses are exacerbated in *Vamp8*^−/−^ mice due to a lack of mucin release.

**FIG 6:**
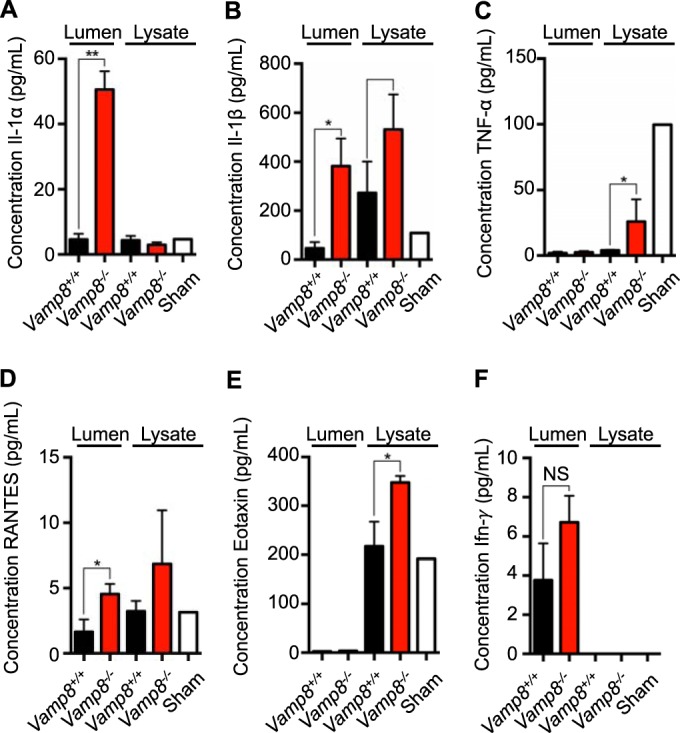
Lack of Vamp8 increases proinflammatory responses *in vivo*. Luminal exudates and full-thickness colonic tissues (lysates) were analyzed by both ELISA and multiplex assay to assess expression levels of (A) Il-1α, (B) IL-1β, (C) TNF-α, (D) RANTES, (E) eotaxin, and (F) IFN-γ following *E. histolytica* infection of colonic loops at 3 h postinfection. *Vamp8*^−/−^ mice showed increased proinflammatory cytokine expression compared to *Vamp8*^*+/+*^ littermates; however, the results were not statistically significant for Th1 cytokines. **, *P* < 0.01; *, *P* < 0.05.

In response to an invading pathogen, the host mounts a proinflammatory response characterized by acute cytokine release. We have previously shown that IL-1α and IL-1β are specifically released by macrophages upon *E. histolytica* contact during *E. histolytica* infection (17, 26). Accordingly, we hypothesized that increased *E. histolytica* contact with the epithelium and apoptosis would result in an exacerbated proinflammatory response in *Vamp8*^−/−^ mice because of the lack of the protective effects of the mucus barrier. Indeed, luminal exudates and tissue lysates from colonic loops infected with *E. histolytica* showed increased IL-1α, IL-1β, and TNF-α levels in *Vamp8*^−/−^ animals compared to *Vamp8*^*+/+*^ littermates (Fig. 6A to C). Interestingly, a significant increase in the levels of RANTES and eotaxin and elevated levels of gamma interferon (IFN-γ) were also seen in *Vamp8*^−/−^ mice in response to *E. histolytica*. Several other cytokines and chemokines were assessed by multiplex assays; however, the differences between the results seen with the *Vamp8*^−/−^ and *Vamp8*^*+/+*^ mice were not significant.

## DISCUSSION

We describe here for the first time how classical exocytosis modulates mucin secretion from intestinal goblet cells. This study focused on the primary R-SNARE VAMP8 present on mucin granules that facilitated mucin exocytosis. Numerous R-SNAREs have been described in mammalian cells, including seven vesicle-associated membrane proteins; however, studies of mast cell degranulation, pancreatic acinar, and islet beta cell insulin exocytosis led to the hypothesis that either VAMP2 or VAMP8 plays a role in nonneuronal exocytosis of mucin (27, 28). In support of this hypothesis, we observed undetectable protein and mRNA levels of VAMP2 and specific localization of VAMP8 to mucin granules. Other studies have alluded to a direct interaction between VAMP8 and the previously identified MARCKS protein present on mucin granules and participating in airway mucus exocytosis (29). Accordingly, this interaction then facilitates constitutive and stimulated release of mucin in the airways through interaction with SNAP23 and Munc18b. We have recently shown that MARCKS was directly phosphorylated and activated on mucin granules following *E. histolytica* CP5 (EhCP5) ligation to integrins (15). Continuation of that work in the current study showed that this pathway precedes VAMP8 mucin granules undergoing exocytosis. Given that many pathogens are likely to activate signaling cascades in various manners, we believe that all the pathways converge on VAMP8-mediated exocytosis. This model of mucin-induced exocytosis therefore has broad application to various pathogens that stimulate mucin secretion. Specifically, mucin has been shown to be critical in limiting *Salmonella* burden and epithelial dysfunction *in vivo* (30). Additionally, several bacteria toxins, including listeriolysin O from *Listeria monocytogenes* and cholera toxin from *Vibrio cholerae*, have been shown to induce mucin secretion by unknown mechanisms (31, 32). Regardless of the presence or absence of calcium- or PKC-dependent signaling, VAMP8 may participate in mucin exocytosis. Numerous studies have demonstrated that both Ca2^+^ and PKC agonists, classically, calcium ionophore and PMA, are potent mucin secretagogues (33). As several SNARE chaperones that mediate R-SNARE complex formation with Q_abc_ SNAREs can be targets of calcium and PKC, this promiscuity of exocytosis activation likely represents a protective mechanism used to deal with a broad range of threats (34).

Breakdown of the mucus barrier has been well described to exacerbate disease and has grave consequences for barrier function basally and in response to various pathogens. In studies using experimental colitis, *Muc2*^−/−^ and Winnie-misfolded Muc2 animals are highly susceptible to dextran sulfate sodium (DSS)-induced colitis (35, 36). Loss of the protective functions of mucin ultimately leads to increased apoptosis of the mucosa and subsequent proinflammatory infiltrate formation. In *E. histolytica* disease pathogenesis, loss of MUC2 during infection leads to tight junction permeability and proinflammatory responses predominantly due to the *E. histolytica* virulence factor EhCP5 (10). Attaching and effacing pathogens such as *Citrobacter rodentium* also cause increased pathogenesis and mortality in mice lacking mucin as demonstrated in *Muc2*^−/−^ animals (9).

Proteomic approaches have elucidated numerous mucin granule-associated proteins within intestinal and airway goblet cells (37, 38). In airway goblet cells, a myriad of actin-associated proteins, including myosin, ARP2/3, and MARCKS, have been identified (39). A plausible hypothesis for this actin dependency is that it could be structural or associated with trafficking or contraction of the goblet cell theca to prime exocytosis. We have observed an actin ring around the theca of mucin granules *in vitro* in support of this hypothesis. Coordination of the activity of HSP70 and cysteine string protein (CSP) in priming of vesicles during exocytosis, thus facilitating protein complex assembly, has also been previously identified (37). In support of the idea of the involvement of classical exocytosis in coordinating mucin secretion, several Rab proteins, including Rab3 and Rab27, that are implicated in exocytosis of neuronal cells have been shown to associate with mucin granules (40). These Rab proteins have a role in participating in calcium-mediated exocytosis, whereby Rab3 has been shown to associate with the Munc13-RIM complex to prime vesicles. These Rab proteins also participate in vesicle trafficking, where Rab3 binds myosin V_a_ for transportation along actin filaments (40). Vamp7 and Vamp8 have been identified on mucin granules in proteomic studies in which both colocalized to mucin granules within the goblet cell theca (38). While VAMP8 was specifically activated in response to *E. histolytica*, the localization around a mucin-organizing center was surprising. VAMP8 colocalized basally to mucin granules as predicted; upon stimulation, however, a supramolecular structure within the theca appeared to form. Here, VAMP8 contacted the plasma membrane and facilitated mucin exocytosis as evidenced by the increase in the luminal pH (5.4 to 7.4) of mucin granules (21). This phenomenon is strikingly similar to the secretion of von Willebrand factor (VWF) from endothelial cells. VWF and mucin possess similar structural components, given that they are both heavily glycosylated and are polymeric. Within endothelial cells, VWF vesicles coalesce into supramolecular cytoplasmic regions called Weibel-Palade bodies destined for exocytosis (41). *In vitro*, our results demonstrated that similar mechanisms are likely involved within goblet cells, thus providing the fast secretion required to fend off a pathogen. *In vivo*, the observation that mucin granules are relatively large (approx 1 μm) could account for granule-granule fusion, as the vesicles that leave the Golgi compartment are clearly not this size. Strikingly, the morphology of mucin granules in *Vamp8*^−/−^ animals is drastically irregular in comparison to the highly ordered “grape” organization in *Vamp*^*+/+*^ littermates. This is likely the result of abolishment of apical exocytosis due to a lack of Vamp8, resulting in mucin granules arrested within the theca and leading to bloating of the goblet cells and coalesced granules. Regardless, if mucin granules form a supramolecular structure or undergo granule-granule fusion, these mechanisms ultimately result in an increase in the speed of exocytosis as the rate-limiting step in exocytosis is the availability of SNARE association on the plasma membrane for docking (40). The presence of larger vesicles inherently increases the rate at which vesicular content can be released.

In the context of *E. histolytica* infection, induced mucus secretion by goblet cells is clearly a protective mechanism to spatially fend off potentially invading parasites. Abolishment of exocytosis in goblet cells by silencing VAMP8 expression or in *Vamp8*^−/−^ animals leads to abrogated mucin secretion and increased *E. histolytica* adherence. We suspect that host killing in epithelial cells is mediated through amoebapore apoptosis, as long-term (>60-min) live-cell imaging revealed membrane blebbing of LS174T cells. It has been well characterized that this process occurs in a caspase 3-dependent manner and follows classical apoptosis leading to DNA fragmentation (42, 43). Indeed, caspase 3 was specifically cleaved both *in vitro* and *in vivo*, and the process culminated in DNA fragmentation detected by TUNEL staining. Trogocytosis has emerged has an alternative method of cell killing; however, we rarely observed trogocytosis in epithelial cells by staining with plasma membrane dyes (44). This result was likely due to the organization of the host cytoskeleton and junctional complexes, which does not permit drastic flexibility in the plasma membrane, especially when LS174T cells are grown in monolayers. Calcium influx induced by *E.  histolytica* has been shown to drive amoebapore-dependent apoptosis in cells (44). Our previous studies showed that at early time points (<10 min), *E.  histolytica* induced little to no influx of calcium in LS174T cells; however, longer durations and higher MOIs have yet to be investigated (15). Additionally, we do not believe that the release of mucin from apoptotic cells as seen with *Vamp8*^−/−^ animals is either functional or relevant. First, in all secretion experiments, lactate dehydrogenase (LDH) levels were measured and were less than 5% due to an optimized MOI of *E. histolytica*. Second, proper secretion of mucin by regulated exocytosis is critical for mucin structure/function (22). Due to improper hydration, vesicle lysis into the luminal milieu does not result in mucin having the same protective features.

A requirement for intestinal amoebiasis is penetration of *E. histolytica* past mucosal epithelial cells and association within the lamina propria (45). Mouse models of *E. histolytica* infection poorly interrogate this event; however, it is clear that impairment of VAMP8 mucin exocytosis exacerbates this event. The resulting proinflammatory responses elicited by the host are likely mediated by macrophages and dendritic cells present in the lamina propria through direct or indirect contact mechanisms. In support of this, we have previously demonstrated that virulence factors EhCP5 and Gal lectin are fundamental in activation of the NLRP3 inflammasome, resulting in IL-1α and IL-1β secretion (17, 26). Indeed, the level of secretion of these inflammasome-dependent cytokines was increased in *Vamp8*^−/−^ animals compared to *Vamp8*^*+/+*^ littermates during acute *E. histolytica* infection. In addition, tumor necrosis factor alpha (TNF-α) secretion has been implicated in worsened disease outcome during *E. histolytica* pathogenesis whereas IFN-γ secretion is protective (45, 46). *Vamp8*^−/−^ acutely exposed to *E. histolytica* showed a slight increase in TNF-α secretion levels and very low levels of IFN-γ (in terms of pictograms per milliliter secreted). This secretion likely occurs through indirect contact of *E. histolytica* with macrophages in the lamina propria, as several amoebic components, including lipopeptidophosphoglycan (LPPG) and *E. histolytica* DNA, can stimulate TNF-α secretion *in vitro* through the presence of Toll-like receptor 2 (TLR2) and TLR4, respectively (47, 48). Secretion of TNF-α and other proinflammatory cytokines may occur through activation of NF-κB by EhCP5 (49). This is also associated with worsened disease outcome, while levels of Th1 cytokines such as IFN-γ and Il-12p40 were low in a previous study (50). Taken together, these results demonstrate that *Vamp8*^−/−^ animals have increased susceptibility to *E. histolytica* infection due to improper mucin exocytosis in goblet cells.

## MATERIALS AND METHODS

### Cell culture and animal studies.

Human adenocarcinoma colonic goblet cells (LS174T [ATCC CL-188]) were subjected to routine passage through nude mice to maintain a high-mucin phenotype (51). The LS174T cells were cultured in Eagle's minimum essential medium (EMEM) supplemented with 10% fetal bovine serum (FBS), 20 mM HEPES, and 100 U/ml penicillin/streptomycin. The LS174T cells were passaged with 0.25% trypsin-EDTA (Thermo) once cells reached 90% confluence. For secretion experiments, LS174T cells were seeded in 24-well plates in triplicate at a density of 5 × 10^4^ and cultured until a confluent monolayer was formed. For biochemical assays, 8 × 10^5^ cells/well were seeded in 6-well plates and allowed to reach 70% confluence. For lentivirus silencing of VAMP8, LS174T cells were seeded in 24-well plates and infected with lentivirus containing transfer plasmid pTRIPZ-VAMP8 (CloneID: V2THS_23739) at an MOI of 10. Transduced cells were routinely sorted by FACS analysis for TurboRFP expression to maintain knockdown. Uninduced and doxycycline (2µg/ml)-treated LS174T cells were cultured separately to suppress expression of VAMP8, which has a long half-life. This also allowed off-target effects of sorting, selection, and lentivirus presence to be controlled for. Additionally, pTRIPZ lentivirus-inducible shRNA control particles (catalog no. RHS4827) were also transduced into LS174T; in all experiments, these behaved identically to the uninduced VAMP8KD cells and the wild-type (WT) LS174T cells. The WT LS174T cells and pTRIPZ control LS174T cells were not affected by the presence of doxycycline with respect to mucin secretion.

Six-to-8-week-old *Vamp8*^*+/+*^ and *Vamp8*^−/−^ littermates on an SV129 background were bred in-house and used for colonic loop infections with *E. histolytica* as previously described (24). Mice were kept in sterilized, filter-top cages and were maintained under specific-pathogen-free conditions with food and water available *ad libitum*. For *E. histolytica* infections, a laparotomy was performed on anesthetized mice to expose the proximal colon, and a colonic loop was created by suturing from the cecum-colon junction 3 cm down the length of the colon. Mice were then infected with 1 × 10^6^ log-phase *E. histolytica* cells mixed with 100 µl of PBS; sham infection animals received only PBS. Infections were carried out for 3 h, with animal welfare carefully monitored. All studies were approved by the University of Calgary Animal Care Committee.

### Cultivation and harvesting of *E. histolytica.*

*E. histolytica* (HM1:IMSS; ATCC) cells were cultured in TYI-S-3 medium containing 100 U/ml penicillin/streptomycin at 37°C under axenic conditions (52). After 72 h, logarithmic-growth-phase *E. histolytica* cultures were harvested by chilling on ice for 9 min, pelleted at 200 × *g*, and washed two times with PBS. *E. histolytica* cells were subjected to routine passage through the liver of gerbils to maintain high virulence (52).

### Reagents and cytokine quantification.

All reagents were purchased from Sigma-Aldrich unless otherwise specified. Bisindolylmaleimide I was purchased from Cayman Chemical. [^3^H]glucosamine was purchased from PerkinElmer. All fluorescent reagents, including WGA-Alexa 647, CFSE, and CellMask Deep Red, and secondary antibodies were from Thermo. Caspase 3, caspase 9, and PARP were from Cell Signaling. Enzyme-linked immunosorbent assay (ELISA) DuoSet kits for IL-1β, IL-1α, and TNF-α as well as the anti-Vamp8 antibody were from R&D. VAMP8-pHluorin was a kind gift from Rytis Prekeris (University of Colorado), and pmRuby2-MUC2CK was synthesized by Genscript as previously described (15, 20, 22). Mouse 41-plex cytokine arrays were constructed by Eve Technologies on the basis of tissue lysates normalized to 1 mg/ml.

### Confocal microscopy.

For colonic loop studies, tissues were fixed in Carnoy's fixative, embedded in paraffin, and sectioned at 7 μm. After rehydration of tissue sections through an ethanol gradient, sections were permeabilized with 0.2% Triton for 5 min, blocked with normal donkey serum, and incubated with an anti-Muc2 antibody (H-300; Santa Cruz) overnight at 4°C. The following day, samples were washed thrice with Tris-PBS (TPBS)–0.2% bovine serum albumin (BSA) and stained with secondary antibodies, DAPI (4′,6-diamidino-2-phenylindole), and WGA-Alexa 647 for 1 h at room temperature. After three washes, sections were mounted in FluoroSave (CalibroChem) and imaged on an Olympus FV1000 confocal scanning microscope. TUNEL staining (ApopTag; Millipore) was carried out according to the instructions of the manufacturer with the inclusion of proteinase K antigen retrieval and Triton permeabilization. Live-cell imaging was performed by transfecting LS174T cells for 24 h with pHluorin-VAMP8 and pmRuby2-MUC2CK using JetPrime. Five minutes prior to imaging, transfected cells were labeled with CellMask Deep Red and subsequently washed. Simultaneously, *E. histolytica* cells were labeled with CellTracker Blue for 30 min at 37C and washed twice with PBS. Transfected LS174T cells were infected with *E. histolytica* at an MOI of 0.2 and continuously imaged for up to 1 h on a Nikon A1R resonant scanner at 37C. Quantification of pHluorin-VAMP8 was done by selecting a region of interest (ROI) containing mucin and plotting GFP intensity in Nikon Elements. Quantification of goblet cell abundance and mucin content from colonic loop still images was done in ImageJ by selecting an ROI containing the epithelium and plotting the total intensity/unit area or by counting DAPI^+^ nuclei and plotting total intensity/average cell intensity. A minimum of 500 cells were analyzed for all quantifications performed with intestinal tissue. Figures were generated in Adobe Photoshop CS5.

### Quantification of mucin secretion.

Newly synthesized LS174T mucin was metabolically labeled with 2 μCi/ml of [^3^H]glucosamine for 24 h prior to the mucin secretion experiments. Cells were washed twice with PBS and media to remove free [^3^H]glucosamine and replaced with serum-free EMEM. For kinase inhibitor experiments, cells were pretreated for 30 min with 10 µM BIM1, and the original media were replaced with fresh media. All secretion experiments were carried out for 2 h at 37°C, after which 80% of the cell supernatant was loaded into scintillation vials for counting. The LS174T cells were infected at an MOI of 0.2 for all secretion experiments. Unless otherwise noted, control baselines were established from untreated WT LS174T cells.

### Immunoblotting.

LS174T cells were washed 3 times with PBS and resuspended in lysis buffer containing 20 mM HEPES, 150 mM NaCl, 1 mM EDTA, 1% NP-40, 10 μM E-64, and protease inhibitor cocktail (Roche) followed by sonication. For tissue lysates, colons were lysed in lysis buffer containing 20 mM Tris-HCl, 0.5% Tween 20, 150 mM NaCl, and protease inhibitor cocktail followed by homogenization and sonication. Protein content was determined by bicinchoninic acid (BCA) assay according to the instructions of the manufacturer (Thermo). A 20-μg volume of protein lysate was suspended with Laemmli sample buffer and boiled with 5% β-mercaptoethanol (BME). Samples were run on reducing 10% polyacrylamide Tris gels and transferred to 0.2-μm-pore-size nitrocellulose. Membranes were blocked with 5% nonfat milk, washed with PBST, and incubated with primary antibodies in PBST–3% BSA overnight at 4°C. The following day, membranes were washed 3 times with PBST and incubated for 1 h at room temperature with secondary horseradish peroxidase (HRP) antibodies–5% nonfat milk followed by detection with ChemiLucent ECL on film.

### *E. histolytica* adherence assay.

LS174T and VAMP8KD cells were seeded in 6-well dishes at a density of 1 × 10^6^ cells/well. After 24 h, cells were labeled with 2.5 μM CFSE for 15 min and washed prior to infection with *E. histolytica* at an MOI of 1. *E. histolytica* cells were labeled with CellMask Deep Red for 5 min at a 1:1,000 dilution followed by washing with PBS. After 30 min of infection, wells were washed twice with PBS to remove nonadherent *E. histolytica* cells and dissociated with trypsin. Cell suspensions were washed with FACS buffer (PBS–2% FBS–2 mM EDTA), filtered through a 100-μm-pore-size filter, and analyzed on a BD Canto system. Unlabeled *E. histolytica* and LS174T cells were used to establish forward scatter/side scatter (FSC/SSC) gating whereby epithelial cells and *E. histolytica* cells scattered distinctly in addition to their respective dyes. Data were analyzed in FlowJo.

### Monolayer destruction assay.

LS174T and VAMP8KD monolayers seeded in 24-well plates were infected with *E. histolytica* at an MOI of 1 for 4 h at 37°C. Wells were washed thrice with PBS and fixed in 2.5% glutaraldehyde for 1 h. Following extensive washing with PBS, cells were stained with 0.1% methylene blue–10 mM borate buffer and washed thrice in borate buffer. Methylene blue was extracted with 0.1 M HCl isopropanol and read at 650 nm in a spectrophotometer.

### Lipid raft flotation.

LS174T cells were seeded into 100-mm-diameter dishes at a density of 2.5 × 10^6^ cells/dish and allowed to reach 90% confluence. Monolayers were infected with *E. histolytica* at an MOI of 0.2 and incubated at 37°C for 2 h. Cells were then washed thrice with PBS and harvested in a buffer containing 25 mM MES (morpholineethanesulfonic acid), 150 mM NaCl, and protease inhibitor cocktails at pH 6.5. Lysis was carried out by passing cells through a Dounce homogenizer 25 times, and the lysate was precleared at 1,000 × *g*. Solubilized cells were titrated to 40% sucrose and overlaid in MES buffer with 30% sucrose followed by 5% sucrose. Ultracentrifugation at 240,000 × *g* in an SW-40 Ti rotor was carried out for 18 h, and 1-ml fractions were collected. Here, the lipid rafts floated to fractions 4 to 5 between the 30% and 5% sucrose layers whereas the nonraft components stayed in the 40% sucrose layer near the bottom of the gradient (fractions 8 to 11).

### Statistics.

All data shown are representative of three independent experiments unless otherwise noted. Data and graphs were plotted in Prism GraphPad, and significance was calculated by either analysis of variance (ANOVA) or the Student *t* test. Statistical significance was defined as *P* values of <0.05, with all results displayed as means ± standard errors of the means (SEM).

## References

[1] Cornick S, Tawiah A, Chadee K 2015 Roles and regulation of the mucus barrier in the gut. Tissue Barriers 3:e982426. doi:10.4161/21688370.2014.982426. 25838985 PMC4372027

[2] Johansson MEV, Larsson JMH, Hansson GC 2011 The two mucus layers of colon are organized by the MUC2 mucin, whereas the outer layer is a legislator of host-microbial interactions. Proc Natl Acad Sci U S A 108(Suppl 1):4659–4665. doi:10.1073/pnas.1006451107. 20615996 PMC3063600

[3] Hollingsworth MA, Swanson BJ 2004 Mucins in cancer: protection and control of the cell surface. Nat Rev Cancer 4:45–60. doi:10.1038/nrc1251. 14681689

[4] Thomsson KA, Holmén-Larsson JM, Angström J, Johansson ME, Xia L, Hansson GC 2012 Detailed O-glycomics of the Muc2 mucin from colon of wild-type, core 1- and core 3-transferase-deficient mice highlights differences compared with human MUC2. Glycobiology 22:1128–1139. doi:10.1093/glycob/cws083. 22581805 PMC3382349

[5] Larsson JM, Karlsson H, Sjövall H, Hansson GC 2009 A complex, but uniform O-glycosylation of the human MUC2 mucin from colonic biopsies analyzed by nanoLC/MSn. Glycobiology 19:756–766. doi:10.1093/glycob/cwp048. 19321523

[6] Chadee K, Petri WA, Innes DJ, Ravdin JI 1987 Rat and human colonic mucins bind to and inhibit adherence lectin of *Entamoeba histolytica*. J Clin Invest 80:1245–1254. doi:10.1172/JCI113199. 2890655 PMC442377

[7] Linden SK, Sutton P, Karlsson NG, Korolik V, McGuckin MA 2008 Mucins in the mucosal barrier to infection. Mucosal Immunol 1:183–197. doi:10.1038/mi.2008.5. 19079178 PMC7100821

[8] Cornick S, Chadee K 2017 *Entamoeba histolytica*: host parasite interactions at the colonic epithelium. Tissue Barriers 5:e1283386. doi:10.1080/21688370.2017.1283386. 28452682 PMC5362996

[9] Bergstrom KSB, Kissoon-Singh V, Gibson DL, Ma C, Montero M, Sham HP, Ryz N, Huang T, Velcich A, Finlay BB, Chadee K, Vallance BA 2010 Muc2 protects against lethal infectious colitis by disassociating pathogenic and commensal bacteria from the colonic mucosa. PLoS Pathog 6:e1000902. doi:10.1371/journal.ppat.1000902. 20485566 PMC2869315

[10] Kissoon-Singh V, Moreau F, Trusevych E, Chadee K 2013 *Entamoeba histolytica* exacerbates epithelial tight junction permeability and proinflammatory responses in Muc2(-/-) mice. Am J Pathol 182:852–865. doi:10.1016/j.ajpath.2012.11.035. 23357502

[11] Specian RD, Neutra MR 1980 Mechanism of rapid mucus secretion in goblet cells stimulated by acetylcholine. J Cell Biol 85:626–640. doi:10.1083/jcb.85.3.626. 7391135 PMC2111470

[12] Birchenough GMH, Johansson MEV, Gustafsson JK, Bergström JH, Hansson GC 2015 New developments in goblet cell mucus secretion and function. Mucosal Immunol 8:712–719. doi:10.1038/mi.2015.32. 25872481 PMC4631840

[13] Südhof TC, Rizo J 2011 Synaptic vesicle exocytosis. Cold Spring Harb Perspect Biol 3. doi:10.1101/cshperspect.a005637. PMC3225952 22026965

[14] Fasshauer D 2003 Structural insights into the SNARE mechanism. Biochim Biophys Acta 1641:87–97. doi:10.1016/S0167-4889(03)00090-9. 12914950

[15] Cornick S, Moreau F, Chadee K 2016 *Entamoeba histolytica* cysteine proteinase 5 evokes mucin exocytosis from colonic goblet cells via αvβ3 integrin. PLoS Pathog 12:e1005579. doi:10.1371/journal.ppat.1005579. 27073869 PMC4830554

[16] Lidell ME, Moncada DM, Chadee K, Hansson GC 2006 *Entamoeba histolytica* cysteine proteases cleave the MUC2 mucin in its C-terminal domain and dissolve the protective colonic mucus gel. Proc Natl Acad Sci U S A 103:9298–9303. doi:10.1073/pnas.0600623103. 16754877 PMC1482604

[17] Mortimer L, Moreau F, Cornick S, Chadee K 2014 Gal-lectin-dependent contact activates the inflammasome by invasive *Entamoeba histolytica*. Mucosal Immunol 7:829–841. doi:10.1038/mi.2013.100. 24253103

[18] Axelsson MA, Asker N, Hansson GC 1998 O-glycosylated MUC2 monomer and dimer from LS 174T cells are water-soluble, whereas larger MUC2 species formed early during biosynthesis are insoluble and contain nonreducible intermolecular bonds. J Biol Chem 273:18864–18870. doi:10.1074/jbc.273.30.18864. 9668062

[19] Belley A, Keller K, Grove J, Chadee K 1996 Interaction of LS174T human colon cancer cell mucins with *Entamoeba histolytica*: an in vitro model for colonic disease. Gastroenterology 111:1484–1492. doi:10.1016/S0016-5085(96)70009-4. 8942726

[20] Marshall MR, Pattu V, Halimani M, Maier-Peuschel M, Müller ML, Becherer U, Hong W, Hoth M, Tschernig T, Bryceson YT, Rettig J 2015 VAMP8-dependent fusion of recycling endosomes with the plasma membrane facilitates T lymphocyte cytotoxicity. J Cell Biol 210:135–151. doi:10.1083/jcb.201411093. 26124288 PMC4493996

[21] Ambort D, Johansson MEV, Gustafsson JK, Nilsson HE, Ermund A, Johansson BR, Koeck PJB, Hebert H, Hansson GC 2012 Calcium and pH-dependent packing and release of the gel-forming MUC2 mucin. Proc Natl Acad Sci U S A 109:5645–5650. doi:10.1073/pnas.1120269109. 22451922 PMC3326483

[22] Perez-Vilar J, Olsen JC, Chua M, Boucher RC 2005 pH-dependent intraluminal organization of mucin granules in live human mucous/goblet cells. J Biol Chem 280:16868–16881. doi:10.1074/jbc.M413289200. 15718243

[23] Salaün C, Gould GW, Chamberlain LH 2005 Lipid raft association of SNARE proteins regulates exocytosis in PC12 cells. J Biol Chem 280:19449–19453. doi:10.1074/jbc.M501923200. 15769746 PMC2394574

[24] Chadee K, Keller K, Forstner J, Innes DJ, Ravdin JI 1991 Mucin and nonmucin secretagogue activity of *Entamoeba histolytica* and cholera toxin in rat colon. Gastroenterology 100:986–997. doi:10.1016/0016-5085(91)90274-O. 2001836

[25] Saffer LD, Petri WA 1991 Role of the galactose lectin of *Entamoeba histolytica* in adherence-dependent killing of mammalian cells. Infect Immun 59:4681–4683. 1937828 10.1128/iai.59.12.4681-4683.1991 PMC259097

[26] Mortimer L, Moreau F, Cornick S, Chadee K 2015 The NLRP3 inflammasome is a pathogen sensor for invasive *Entamoeba histolytica* via activation of α5β1 integrin at the macrophage-amebae intercellular junction. PLOS Pathog 11:e1004887. doi:10.1371/journal.ppat.1004887. 25955828 PMC4425650

[27] Wang CC, Ng CP, Lu L, Atlashkin V, Zhang W, Seet LF, Hong W 2004 A role of VAMP8/endobrevin in regulated exocytosis of pancreatic acinar cells. Dev Cell 7:359–371. doi:10.1016/j.devcel.2004.08.002. 15363411

[28] Lorentz A, Baumann A, Vitte J, Blank U 2012 The SNARE machinery in mast cell secretion. Front Immunol 3:143. doi:10.3389/fimmu.2012.00143. 22679448 PMC3367400

[29] Adler KB, Tuvim MJ, Dickey BF 2013 Regulated mucin secretion from airway epithelial cells. Front Endocrinol (Lausanne) 4:129. doi:10.3389/fendo.2013.00129. 24065956 PMC3776272

[30] Zarepour M, Bhullar K, Montero M, Ma C, Huang T, Velcich A, Xia L, Vallance BA 2013 The mucin Muc2 limits pathogen burdens and epithelial barrier dysfunction during *Salmonella enterica* serovar Typhimurium colitis. Infect Immun 81:3672–3683. doi:10.1128/IAI.00854-13. 23876803 PMC3811786

[31] Coconnier MH, Dlissi E, Robard M, Laboisse CL, Gaillard JL, Servin AL 1998 *Listeria monocytogenes* stimulates mucus exocytosis in cultured human polarized mucosecreting intestinal cells through action of listeriolysin O. Infect Immun 66:3673–3681. 9673248 10.1128/iai.66.8.3673-3681.1998 PMC108401

[32] Epple HJ, Kreusel KM, Hanski C, Schulzke JD, Riecken EO, Fromm M 1997 Differential stimulation of intestinal mucin secretion by cholera toxin and carbachol. Pflugers Arch 433:638–647. doi:10.1007/s004240050325. 9049150

[33] Dray-Charier N, Paul A, Combettes L, Bouin M, Mergey M, Balladur P, Capeau J, Housset C 1997 Regulation of mucin secretion in human gallbladder epithelial cells: predominant role of calcium and protein kinase C. Gastroenterology 112:978–990. doi:10.1053/gast.1997.v112.pm9041261. 9041261

[34] Lee M, Chung S, Uhm DY, Park MK 2005 Regulation of zymogen granule exocytosis by Ca2+, cAMP, and PKC in pancreatic acinar cells. Biochem Biophys Res Commun 334:1241–1247. doi:10.1016/j.bbrc.2005.07.015. 16040001

[35] Van der Sluis M, De Koning BAE, De Bruijn ACJM, Velcich A, Meijerink JPP, Van Goudoever JB, Büller HA, Dekker J, Van Seuningen I, Renes IB, Einerhand AWC 2006 Muc2-deficient mice spontaneously develop colitis, indicating that MUC2 is critical for colonic protection. Gastroenterology 131:117–129. doi:10.1053/j.gastro.2006.04.020. 16831596

[36] Cobo ER, Kissoon-Singh V, Moreau F, Holani R, Chadee K 2017 MUC2 mucin and butyrate contribute to the synthesis of the antimicrobial peptide cathelicidin in response to *Entamoeba histolytica* and dextran sodium sulfate-induced colitis. Infect Immun 85:00905–00916. doi:10.1128/IAI.00905-16. PMC5328487 28069814

[37] Raiford KL, Park J, Lin KW, Fang S, Crews AL, Adler KB 2011 Mucin granule-associated proteins in human bronchial epithelial cells: the airway goblet cell “granulome”. Respir Res 12:118. doi:10.1186/1465-9921-12-118. 21896166 PMC3184067

[38] Rodríguez-Piñeiro AM, van der Post S, Johansson MEV, Thomsson KA, Nesvizhskii AI, Hansson GC 2012 Proteomic study of the mucin granulae in an intestinal goblet cell model. J Proteome Res 11:1879–1890. doi:10.1021/pr2010988. 22248381 PMC3292267

[39] Li Y, Martin LD, Spizz G, Adler KB 2001 MARCKS protein is a key molecule regulating mucin secretion by human airway epithelial cells in vitro. J Biol Chem 276:40982–40990. doi:10.1074/jbc.M105614200. 11533058

[40] Cazares VA, Subramani A, Saldate JJ, Hoerauf W, Stuenkel EL 2014 Distinct actions of Rab3 and Rab27 GTPases on late stages of exocytosis of insulin. Traffic 15:997–1015. doi:10.1111/tra.12182. 24909540 PMC4140954

[41] Valentijn KM, van Driel LF, Mourik MJ, Hendriks GJ, Arends TJ, Koster AJ, Valentijn JA 2010 Multigranular exocytosis of Weibel-Palade bodies in vascular endothelial cells. Blood 116:1807–1816. doi:10.1182/blood-2010-03-274209. 20448112

[42] Ragland BD, Ashley LS, Vaux DL, Petri WA 1994 *Entamoeba histolytica*: target cells killed by trophozoites undergo DNA fragmentation which is not blocked by Bcl-2. Exp Parasitol 79:460–467. doi:10.1006/expr.1994.1107. 7957763

[43] Huston CD, Houpt ER, Mann BJ, Hahn CS, Petri WA 2000 Caspase 3-dependent killing of host cells by the parasite *Entamoeba histolytica*. Cell Microbiol 2:617–625. doi:10.1046/j.1462-5822.2000.00085.x. 11207613

[44] Ralston KS, Solga MD, Mackey-Lawrence NM, Somlata, Bhattacharya A, Petri WA 2014 Trogocytosis by *Entamoeba histolytica* contributes to cell killing and tissue invasion. Nature 508:526–530. doi:10.1038/nature13242. 24717428 PMC4006097

[45] Mortimer L, Chadee K 2010 The immunopathogenesis of *Entamoeba histolytica*. Exp Parasitol 126:366–380. doi:10.1016/j.exppara.2010.03.005. 20303955

[46] Ankri S 2015 *Entamoeba histolytica*—tumor necrosis factor: a fatal attraction. Microb Cell 2:216–218. doi:10.15698/mic2015.07.216. 28357296 PMC5349168

[47] Maldonado-Bernal C, Kirschning CJ, Rosenstein Y, Rocha LM, Rios-Sarabia N, Espinosa-Cantellano M, Becker I, Estrada I, Salazar-González RM, López-Macías C, Wagner H, Sánchez J, Isibasi A 2005 The innate immune response to *Entamoeba histolytica* lipopeptidophosphoglycan is mediated by Toll-like receptors 2 and 4. Parasite Immunol 27:127–137. doi:10.1111/j.1365-3024.2005.00754.x. 15910421

[48] Ivory CPA, Prystajecky M, Jobin C, Chadee K 2008 Toll-like receptor 9-dependent macrophage activation by *Entamoeba histolytica* DNA. Infect Immun 76:289–297. doi:10.1128/IAI.01217-07. 17984204 PMC2223673

[49] Hou Y, Mortimer L, Chadee K 2010 *Entamoeba histolytica* cysteine proteinase 5 binds integrin on colonic cells and stimulates NFkappaB-mediated pro-inflammatory responses. J Biol Chem 285:35497–35504. doi:10.1074/jbc.M109.066035. 20837477 PMC2975174

[50] Guo X, Stroup SE, Houpt ER 2008 Persistence of Entamoeba histolytica infection in CBA mice owes to intestinal IL-4 production and inhibition of protective IFN-gamma. Mucosal Immunol 1:139–146. doi:10.1038/mi.2007.18. 19079171

[51] Siddiqui B, Byrd JC, Fearney FJ, Kim YS 1989 Comparison of metabolically labeled mucins of LS174T human colon cancer cells in tissue culture and xenograft. Tumour Biol 10:83–94. doi:10.1159/000217600. 2734549

[52] Clark CG, Diamond LS 2002 Methods for cultivation of luminal parasitic protists of clinical importance. Clin Microbiol Rev 15:329–341. doi:10.1128/CMR.15.3.329-341.2002. 12097242 PMC118080

